# Inhibition of GLO1 in Glioblastoma Multiforme Increases DNA-AGEs, Stimulates RAGE Expression, and Inhibits Brain Tumor Growth in Orthotopic Mouse Models

**DOI:** 10.3390/ijms19020406

**Published:** 2018-01-30

**Authors:** Rahul Jandial, Josh Neman, Punnajit P. Lim, Daniel Tamae, Claudia M. Kowolik, Gerald E. Wuenschell, Sarah C. Shuck, Alexandra K. Ciminera, Luis R. De Jesus, Ching Ouyang, Mike Y. Chen, John Termini

**Affiliations:** 1 Division of Neurosurgery, City of Hope & Beckman Research Institute, 1500 East Duarte Road, Duarte, CA 91010, USA; rjandial@coh.org (R.J.); yebrahim@usc.edu (J.N.); mchen@coh.org (M.Y.C.); 2 Department of Neurosurgery, Keck School Medicine, University of Southern California, 1975 Zonal Ave., Los Angeles, CA 90033, USA; 3 Department of Molecular Medicine, City of Hope & Beckman Research Institute, 1500 East Duarte Road, Duarte, CA 91010, USA; plim@coh.org (P.P.L.); daniel.tamae@csun.edu (D.T.); ckowolik@coh.org (C.M.K.); gwuenschell@coh.org (G.E.W.); sshuck@coh.org (S.C.S.); aciminera@coh.org (A.K.C.); luis.dejesus@chem.tamu.edu (L.R.D.J.); 4 Irell and Manella Graduate School of Biological Sciences, City of Hope & Beckman Research Institute, 1500 East Duarte Road, Duarte, CA 91010, USA; 5 Department of Chemistry and Biochemistry, California State University, Northridge, 18111 Nordhoff Street, Northridge, CA 91330, USA; 6 Department of Chemistry, Texas A&M University, PO Box 30012, College Station, TX 77842, USA; 7 Center for Informatics, City of Hope & Beckman Research Institute, 1500 East Duarte Road, Duarte, CA 91010, USA; couyang@coh.org

**Keywords:** glyoxalase 1, AGEs, RAGE, methylglyoxal, CEdG

## Abstract

Cancers that exhibit the Warburg effect may elevate expression of glyoxylase 1 (GLO1) to detoxify the toxic glycolytic byproduct methylglyoxal (MG) and inhibit the formation of pro-apoptotic advanced glycation endproducts (AGEs). Inhibition of GLO1 in cancers that up-regulate glycolysis has been proposed as a therapeutic targeting strategy, but this approach has not been evaluated for glioblastoma multiforme (GBM), the most aggressive and difficult to treat malignancy of the brain. Elevated GLO1 expression in GBM was established in patient tumors and cell lines using bioinformatics tools and biochemical approaches. GLO1 inhibition in GBM cell lines and in an orthotopic xenograft GBM mouse model was examined using both small molecule and short hairpin RNA (shRNA) approaches. Inhibition of GLO1 with *S*-(*p*-bromobenzyl) glutathione dicyclopentyl ester (*p-*BrBzGSH(Cp)_2_) increased levels of the DNA-AGE *N*^2^-1-(carboxyethyl)-2′-deoxyguanosine (CEdG), a surrogate biomarker for nuclear MG exposure; substantially elevated expression of the immunoglobulin-like receptor for AGEs (RAGE); and induced apoptosis in GBM cell lines. Targeting GLO1 with shRNA similarly increased CEdG levels and RAGE expression, and was cytotoxic to glioma cells. Mice bearing orthotopic GBM xenografts treated systemically with *p*-BrBzGSH(Cp)_2_ exhibited tumor regression without significant off-target effects suggesting that GLO1 inhibition may have value in the therapeutic management of these drug-resistant tumors.

## 1. Introduction

According to the World Health Organization (Geneva, Switzerland), Grade IV glioblastoma multiforme (GBM) is one of the most aggressive and clinically challenging cancers of the central nervous system [1]. The median survival for GBM is typically a dismal 12–14 months, even with surgery, radiation, and chemotherapy [2]. This cancer is marked by invariable recurrence, which underscores its reliance on multiple neoplastic processes. GBMs may display the Warburg effect as a metabolic adaptation for survival advantage and therapeutic resistance. This metabolic remodeling from predominantly mitochondrial oxidative phosphorylation (OXPHOS) towards cytoplasmic glycolysis may promote tumor proliferation within the hypoxic tumor microenvironment while taking advantage of elevated glucose flux within the neural niche [3,4].

The enhanced uptake of glucose by cancer cells is facilitated by elevated expression of the glucose transporters such as glucose transporter 1 (GLUT1) [5]. This avidity has been exploited to image solid tumors in humans using 2-^18^fluoro-2′-deoxy-d-glucose (^18^FDG) in conjunction with positron emission tomography (PET) [6]. Recently, the use of proton magnetic resonance spectroscopy (^1^H-MRS) to directly measure the metabolic byproducts of tumor metabolism has become available to patients. These clinical modalities provide non-invasive metabolic profiling of tumors, and offer great potential for evaluating the efficacy of experimental agents that disrupt metabolic adaptations for therapeutic goals [7].

By significantly increasing their glucose uptake, cancer cells compensate for the relatively low energy conversion rate of glycolysis compared to OXPHOS (2 vs. 36 ATP molecules per glucose, respectively), to provide the necessary carbon substrates required to sustain the up-regulated synthesis of nucleotides, proteins, and lipids [8]. However, increased uptake of glucose results in the incidental accumulation of toxic α-oxo-aldehyde byproducts, including methylglyoxal (MG), glyoxal, and 3-deoxyglucosone (3-DG) [9]. If not rapidly removed, these highly reactive electrophiles can covalently modify proteins, lipids, and DNA to form advanced glycation end-products (AGEs), inactivating or modifying their biological function. AGEs may also activate multifunctional signaling pathways by binding to the immunoglobulin-like membrane receptor for AGEs (RAGE) [10] or by RAGE-independent mechanisms [11]. In RAGE-mediated mechanisms, signal transduction requires the presence of the ligand-binding VC1 domain as well as a c-terminal cytosolic domain (full length isoform, fl-RAGE). Attenuation of signaling can occur via soluble RAGE isoforms, which competitively bind ligands but cannot engage signaling pathways since they lack the c-terminal domain. These soluble forms can arise from alternative splicing (esRAGE) or may be produced extracellularly by proteolytic cleavage of fl-RAGE by the ADAM10 sheddase (sRAGE or cRAGE) [12,13]. Functional receptors must pre-assemble as a multimeric complex within the cell membrane [14]. The soluble RAGE isoforms can compete for ligand binding with fl-RAGE, and/or populate the RAGE multimer with inactive isoforms to inhibit RAGE signaling. This “decoy receptor” function may constitute part of the anti-apoptotic arsenal employed by some cancer cells and potentially explain the abundance of soluble RAGE isoforms reported in certain aggressive tumors [15,16]. 

Limiting the accumulation of AGEs via enhanced detoxification of MG is an additional metabolic adaptation for cancers exploiting the Warburg effect. This anti-apoptotic defense is primarily accomplished via the upregulation of the glyoxalase system (GLO1/GLO2), which converts MG to d-lactate using glutathione (GSH) as a catalytic co-factor [17]. GLO1 has been reported to be amplified at the DNA, RNA, or protein level in a variety of primary human cancers and derived cell lines [18,19,20,21,22]. However, it remains unclear whether GLO1 is overexpressed in GBM [19,21]. If GLO1 is elevated relative to surrounding neural tissue, this would suggest that treatment with GLO1 inhibitors either alone or in combination with other drugs may offer potential benefit in the management of these therapeutically recalcitrant tumors, particularly given the known association of GLO1 overexpression with chemotherapy resistance [23,24].

To evaluate GLO1 in GBM, we examined data from The Cancer Genome Atlas (TCGA) and performed gene and protein expression analyses from surgical GBM specimens and glioma cell lines (U87, T98). These studies revealed enhanced GLO1 expression relative to normal brain. As a result of increased intracellular accumulation of MG in U87 and T98 gliomas following GLO1 inhibition by a small molecule inhibitor or short hairpin RNAs (shRNAs), we observed increased levels of DNA-AGEs in genomic DNA as measured by LC-MS/MS [25]. The measurement of stable surrogate biomarkers of MG exposure, such as *N*^2^-1-(carboxyethyl)-2′-deoxyguanosine (CEdG), is proposed as a method for assessing the efficiency of targeted GLO1 inhibition in vitro and in vivo. Significant increases in RAGE were also observed in U87 and T98 cells upon GLO1 inhibition. Finally, treatment of mice possessing actively growing orthotopic glioma xenografts with the GLO1 inhibitor *S*-(*p*-bromobenzyl) glutathione dicyclopentyl ester (*p-*BrBzGSH(Cp)_2_) resulted in significantly reduced tumor growth with limited off-target effects in normal brain.

## 2. Results

### 2.1. GLO1 and RAGE Are Overexpressed in GBM

**Figure 1:**
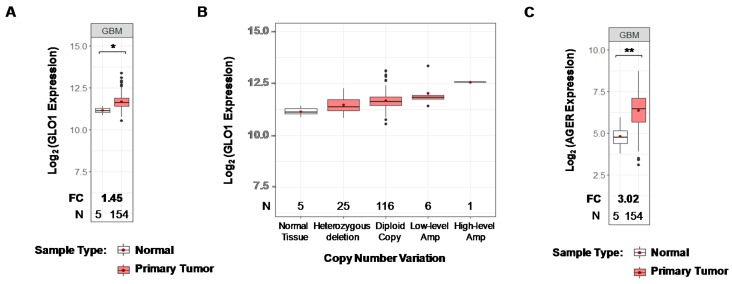
Glyoxylase 1 (GLO1) and RAGE up-regulation in GBM. (**A**) Analyses of TCGA Pan-Cancer project RNA-Seq data for GLO1 (6p21.3-p21.1) in 154 GBMs indicated increased mRNA expression relative to normal brain tissues (*n =* 5). Standard box-plots were applied to visualize the expression distribution for each sample type. The red dots show the average values of each distribution. Fold changes (FC) in gene expression and number of samples (N) are indicated at the bottom. Statistical *p*-values between groups were calculated using Welch’s *t*-test; ** *p* < 0.01, * *p* < 0.05; (**B**) GLO1 mRNA expression in GBM correlates with alterations in gene copy number as determined by GISTIC2 analysis. Expression of GLO1 in normal tissues was plotted as reference. Heterozygous deletion: loss of one copy; Low-level amplification: gain of one extra copy; High-level amplification: gain of two or more extra copies; (**C**) Increased expression (three-fold) of RAGE (AGER) mRNA in GBM relative to normal brain.

GLO1 overexpression is evident in many cancer types, with increases typically ≤2-fold relative to normal tissue (Figure S1). In light of the few scattered and inconsistent reports on GLO1 expression in GBM [19,21], data from The Cancer Genome Atlas (TCGA) Pan-Cancer project RNA-Seq gene expression data was interrogated [26]. For GBM, a 1.5-fold increase of GLO1 mRNA expression was observed relative to normal brain tissues (Figure 1A). The overexpression in GBM was partly contributed by increased gene copy number based on samples having both integrative GLO1 copy number and RNA-Seq expression data. As shown in Figure 1B, a positive correlation between copy number alterations and GLO1 mRNA expression was observed, and 5% (7 of 148) of GBM samples were inferred with copy number gain. Notably, no GLO1 somatic point mutations were found in mutation profiles of 290 samples, indicating a high level of sequence conservation.

The implication of GLO1 overexpression in brain tumors to patients’ outcomes was examined using PROGgeneV2, a web tool for querying prognostic implications of genes in various cancers [27]. A total of thirteen brain cancer datasets were investigated for overall survival analysis, including GBM (*n =* 5), high- or low-grade gliomas (*n =* 5), medulloblastoma (*n =* 2) and meninglioma (*n =* 1). Although the majority of datasets (11 of 13) did not show statistically significant difference in survival outcomes, two NCBI Gene Expression Omnibus (GEO) datasets revealed that high GLO1 expression group was associated with poor survival (GSE13041: GBM; GSE4271: high-grade glioma) [28,29,30]. The overall survival analyses for these two datasets were validated. Kaplan–Meier curves representing high and low GLO1 mRNA expression groups are shown in Figure S2. Although the prognostic implication was not consistently observed across all datasets, the results suggest that GLO1 overexpression may contribute to worse clinical outcomes in certain high-grade glioma cases.

RAGE is typically expressed at low or undetectable levels in normal tissue, with exceptions including lung pneumocytes and CNS cells during development [10,31,32]. However, upon initiation of an inflammatory stimulus, RAGE is strongly expressed [32,33,34]. Due to the recognized role of inflammation in carcinogenesis, RAGE expression has been described in a number of cancers [15]. Analysis of TCGA RNA-Seq data for RAGE (AGER) mRNA expression in 154 GBMs revealed a three-fold average elevated expression relative to normal brain tissues (Figure 1C). A list of other cancers from TCGA Pan-Cancer project that display elevated RAGE is shown in Figure S3.

**Figure 2:**
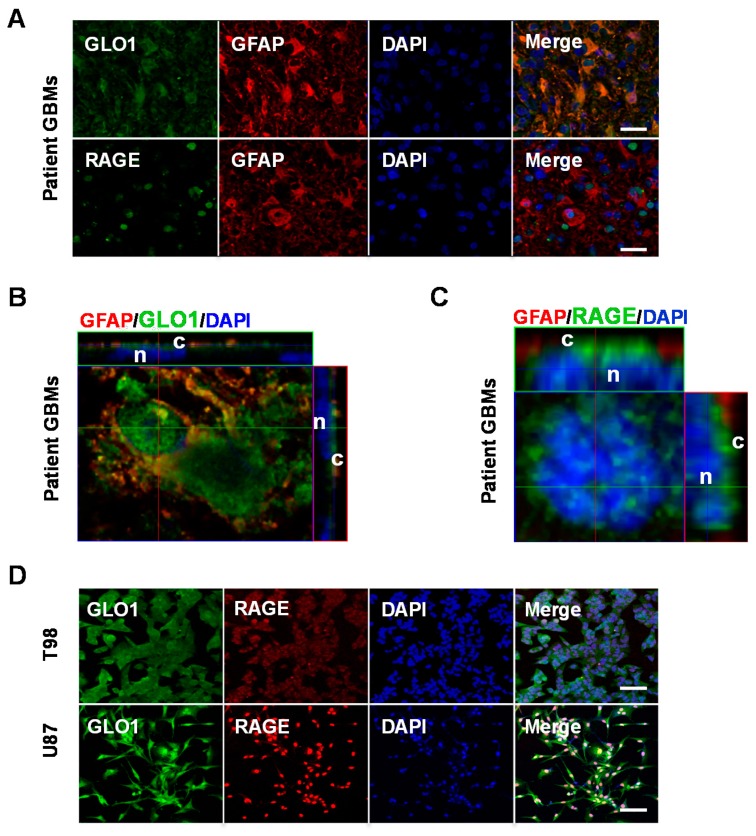

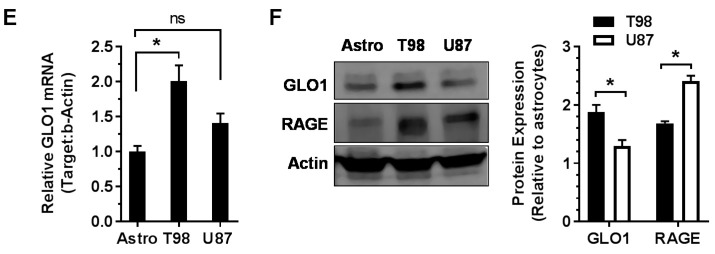
Expression of GLO1 and RAGE in patient GBM tissues and cell lines. (**A**) Immunofluorescence in patient tumor tissue revealed strong GLO1 and RAGE protein expression (green). GFAP staining (red) indicates tumors of glial origin. Nuclei were stained with DAPI (blue); (**B**) Orthogonal view through a confocal *Z*-stack of patient GBM tumor tissue reveals cytoplasmic (c) localization of GLO1 (green) and GFAP (red). Nuclei (n) were stained with DAPI (blue); (**C**) Confocal *Z*-stack analysis of RAGE (green) in patient GBM tissue revealed both cytoplasmic (c) and nuclear/perinuclear (n) localization; (**D**) Immunocytochemistry of T98 and U87 GBM cells revealed co-expression of GLO1 (green) and RAGE (red) protein. Nuclei were stained with DAPI (blue). Scale bars represent 40 µm; (**E**) GLO1 mRNA expression levels in T98 and U87 GBM cells were determined by RT-qPCR, normalized to β-actin and compared to astrocyte controls; (**F**) Western blot analysis and quantification revealed GLO1 and RAGE protein levels in T98 and U87 cells relative to astrocytes; * *p* < 0.05, ns = not significant by Student’s *t*-test.

The expression of GLO1 and RAGE protein in newly obtained human GBM specimens was examined by immunohistochemical analysis (Figure 2A). Tumor pathology was initially confirmed by hematoxylin and eosin (H&E) staining (Figure S4), which revealed scattered pleomorphic nuclei consistent with the pathological criteria for malignancy. The glial origin of the patient samples was confirmed by staining for the cytoskeletal intermediate filament marker glial fibrillary acidic protein (GFAP, Figure 2A). Strong expression of GLO1 and RAGE protein was observed in all patient GBMs that displayed GFAP staining (*n =* 4; Figure 2A (GBM 1) and Figure S5 (GBMs 2–4)). To examine the intracellular distribution of GLO1 in patient derived GBM, an orthogonal view from a confocal *Z*-stacked image was obtained, which revealed expression of GLO1 and GFAP predominantly confined to the cytosol (Figure 2B). In contrast, similar imaging analysis for RAGE protein staining in patient GBMs revealed apparent nuclear/perinuclear accumulation in addition to cytoplasmic localization (Figure 2C).

We next examined mRNA expression and protein levels of GLO1 and RAGE in GBM-derived cell lines T98 and U87. Immunocytochemical staining revealed strong expression of GLO1 and RAGE protein in both cell lines (Figure 2D). Measurement of GLO1 mRNA by RT-qPCR revealed modest up-regulation in both T98 (two-fold, * *p* < 0.05) and U87 (1.4-fold) relative to normal glial cells (astrocytes, Figure 2E). Consistent with this expression pattern, Western blots revealed 1.9- and 1.3-fold increases in GLO1 protein for T98 and U87 cells, respectively (Figure 2F). Relative to astrocytes, T98 and U87 cells displayed 1.7- and 2.4-fold increases in total RAGE protein levels, respectively (Figure 2F).

### 2.2. Small Molecule Inhibition of GLO1 Is Cytotoxic and Induces Apoptosis

**Figure 3:**
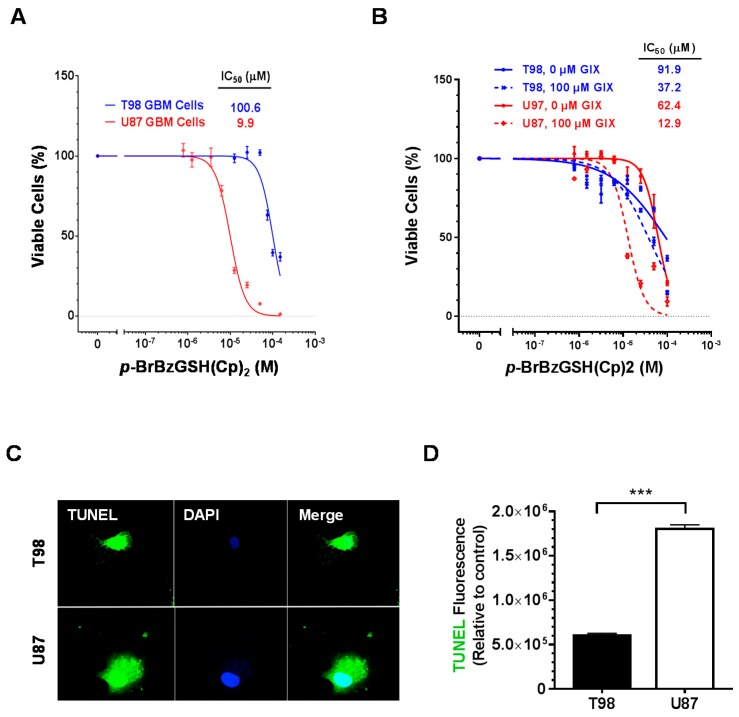
GLO1 inhibition triggered cell death and apoptosis in GBM cells. (**A**) IC_50_ determination following 24 h treatment of U87 and T98 GBM cells with *p*-BrBzGSH(Cp)_2_; (**B**) IC_50_ curves for treatment of T98 and U87 GBM cells with *p*-BrBzGSH(Cp)_2_ following 24 h pretreatment with ADAM10 inhibitor GI254023X (100 µM GIX) or control (0 µM GIX); (**C**) TUNEL-FITC and DAPI imaging of T98 and U87 GBM after treatment with 30 µM *p*-BrBzGSH(Cp)_2_ for 24 h; (**D**) Quantification of TUNEL fluorescence in *p-*BrBzGSH(Cp)_2_ treated T98 and U87 cells normalized to untreated controls. *** *p* < 0.001. Error bars indicate the mean ± SEM.

The effects of GLO1 inhibition in GBM cell lines was examined by treating T98 and U87 cells in culture with the small molecule inhibitor *p*-BrBzGSH(Cp)_2_ [35,36]. Administration of *p-*BrBzGSH(Cp)_2_ was cytotoxic to both cell lines with T98 displaying ~10-fold greater drug resistance relative to U87 (IC_50_ of 100.6 vs. 9.9 µM, respectively, 24 h; Figure 3A). In astrocytes, non-cancer control cells, treatment with *p*-BrBzGSH(Cp)_2_ resulted in ~3-fold lower toxicity relative to the drug-sensitive U87 cells (IC_50_ of 28.9 µM, 24 h). To determine whether cytotoxicity associated with GLO1 inhibition involved an apoptotic response, T98 and U87 GBM cells were treated with 30 µM *p-*BrBzGSH(Cp)_2_ for 24 h and DNA fragmentation was assayed by terminal deoxynucleotidyl transferase dUTP nick end-labeling (TUNEL). Extensive DNA fragmentation in both cell lines following drug treatment was observed (Figure 3C). A three-fold higher level of dUTP labeling at nicked sites was observed in U87 relative to T98 cells suggesting greater sensitivity to MG-induced apoptosis resulting from GLO1 inhibition (Figure 3D).

### 2.3. Inhibition of ADAM10 Increases Sensitivity to p-BrBzGSH(Cp)_2_ and Implicates Partial Protection by sRAGE

Expression of sRAGE has been proposed to play a “decoy receptor” role by interfering with fl-RAGE signaling, either by competitive and non-productive ligand binding or by interfering with assembly of a functional receptor complex in the plasma membrane [16]. Therefore, depletion of sRAGE would be predicted to enhance signaling by fl-RAGE. This was investigated by small molecule inhibition of the metalloproteinase ADAM10, which generates sRAGE via proteolysis of fl-RAGE at the membrane surface [12,13]. Pretreatment of U87 and T98 cells for 24 h with 100 µM of ADAM 10 inhibitor GI254023X followed by administration of *p*-BrBzGSH(Cp)_2_ for an additional 24 h resulted in 4.8- and 2.5-fold decreases, respectively, in the IC_50_ values relative to treatment with GLO1 inhibitor alone (Figure 3B). Treatment of GBM cells with 100 µM of GI254023X alone for 24 h in the absence of GLO1 inhibitor was not cytotoxic (data not shown). These results suggest that sRAGE maturation from the plasma membrane by metalloproteinases partially protects U87 and T98 cells against the cytotoxic effects of GLO1 inhibition.

### 2.4. GLO1 Inhibition Increases Nuclear MG and DNA-AGE Levels

A direct method for validating the targeting efficiency of drugs that inhibit GLO1 would ideally consist of measurement of the dose-dependent accumulation of MG in cell culture or in vivo. Although methods for determining MG levels in biological samples have been described, its highly reactive nature renders direct measurement somewhat unreliable and error prone [37,38]. In contrast, many reaction products of MG with proteins and DNA are thermodynamically stable and may be measured as surrogate biomarkers of MG exposure by appropriate analytical methods. While many amino acid adducts resulting from the reaction of MG with proteins have been described [39], reactions with DNA occur predominantly, if not exclusively, at 2′-deoxyguanosine (dG) [40]. This observation in addition to the fact that all nucleated cells possess the same DNA content suggests that measurement of MG-dG adducts may provide a quantitative indicator of local (tissue DNA) or systemic (blood, urine) exposure to MG. The major thermodynamically stable DNA-AGE in biological samples resulting from reaction with MG is CEdG; we and others have proposed it as a general indicator of carbonyl stress and a specific surrogate biomarker for MG exposure [25,39,41,42,43]. We have described a quantitative and sensitive Liquid Chromatography Electrospray Ionization Tandem Mass Spectrometry (LC-ESI-MS/MS) method using isotope dilution for the measurement of CEdG in biological samples [25]. CEdG was shown to be present in normal tissue at a frequency of 2 per 10^6^ dG, and was also quantifiable in plasma and urine [25]. Measurement of CEdG in DNA before and after treatment with GLO1 inhibitor was used to ascertain the relative efficacy of drug targeting, since inhibition would be predicted to increase levels of MG adducts including DNA-AGEs. 

**Figure 4:**
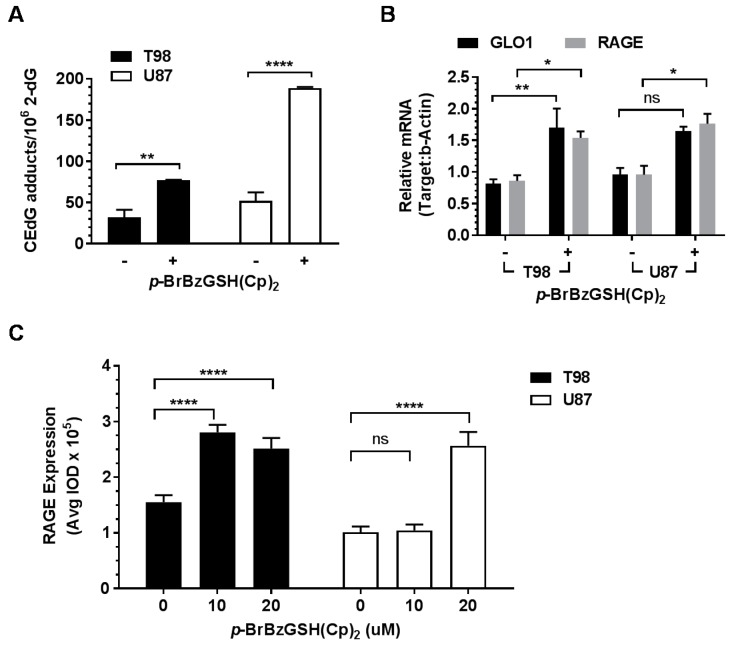

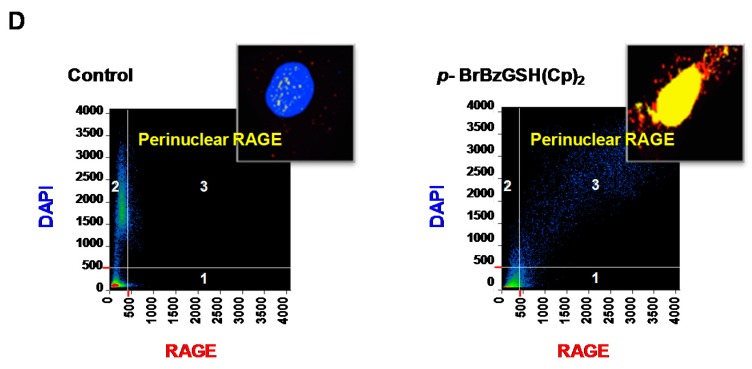
GLO1 inhibition induced RAGE expression and increased DNA-AGEs in GBM cells. (**A**) MS/MS quantification of (***R***, ***S***) CEdG in T98 and U87 GBM cell lines before (−) and after (+) *p-*BrBzGSH(Cp)_2_ treatment (75 and 30 µM, 24 h, respectively). Error bars indicate the mean ± SEM. Data are representative of at least three independent determinations. (**B**) Expression levels of GLO1 and RAGE mRNA in T98 and U98 cells before (−) and after (+) *p-*BrBzGSH(Cp)_2_ treatment (75 and 30 µM, 24 h, respectively). Data represent the mean of at least three independent determinations by RT-qPCR and normalized to β-actin; (**C**) Quantification of RAGE protein in T98 and U87 GBM cells by immunocytochemical staining following 24 h treatment with *p*-BrBzGSH(Cp)_2_, *n* ≥ 50 cells evaluated per condition. IOD = integrated optical density. **** *p* <0.0001, ** *p* < 0.01, * *p* < 0.05, ns = not significant, by two-way analysis of variance (ANOVA). P values were determined using Tukey’s method when correcting for multiple comparisons; (**D**) Representative images for colocalization studies in control and GLO1 inhibitor treated T98 and U87 cells revealed intracellular distribution of RAGE: Gate 1, cytoplasmic RAGE (red); Gate 2, nuclei only (blue); Gate 3, perinuclear RAGE. T98 control cells and inset shows cytoplasmic RAGE distribution (red) and scattered perinuclear staining (yellow) for RAGE. T98 cells treated with *p-*BrBzGSH(Cp)_2_ displayed enhanced perinuclear RAGE and inset shows intense RAGE staining (yellow) within the perinuclear region.

GBM cell lines were treated with *p-*BrBzGSH(Cp)_2_ for 24 h prior to CEdG measurement. CEdG was quantified by mass spectrometry in multiple reaction mode (MRM), monitoring transitions at *m*/*z* 345 → 229 and *m*/*z* 340 → 224 for ^15^N_5_ and ^14^N_5_ CEdG, respectively [25,44] (Figure S6). Prior to LC-ESI-MS/MS analyses, DNA hydrolysates were spiked with (***R***,***S***) ^15^N_5_-CEdG, and ion intensities were fitted to a standard curve. DNA from untreated T98 and U87 cells was analyzed to measure endogenous background levels of CEdG. Levels of (***R***) and (***S***) CEdG diastereomers were measured independently and were summed for each cell line to provide total values for CEdG (Figure 4A). The endogenous CEdG levels in T98 and U87 cells were ~30 and ~50 per 10^6^ dG, respectively. T98 and U87 cells treated with *p-*BrBzGSH(Cp)_2_ at concentrations near the IC_50_ value exhibited two- and four-fold increases in CEdG relative to untreated controls (*p* < 0.01 and *p* < 0.0001, respectively). These findings were consistent with elevated intracellular MG levels as a result of GLO1 inhibition and suggest that the greater sensitivity of U87 cells to *p-*BrBzGSH(Cp)_2_ may be due in part to increased DNA and likely protein modification by MG.

### 2.5. p-BrBzGSH(Cp)_2_ Treatment Induces Expression of GLO1 and RAGE

Because MG administration has been reported to increase the expression of GLO1 and RAGE [45,46], we examined whether increased MG from *p*-BrBzGSH(Cp)_2_ treatment in GBM cells would mimic the effect of direct MG administration observed in other cell types. Immunocytochemistry and RT-qPCR were used to evaluate protein and mRNA expression following treatment with GLO1 inhibitor. GLO1 and RAGE mRNA levels increased 2- and 1.7-fold, respectively, in T98 cells following *p*-BrBzGSH(Cp)_2_ treatment (Figure 4B), while corresponding increases of 1.7- and 1.9-fold were observed in U87 cells. Immunocytochemistry and integration of RAGE immunofluorescence prior to and 24 h after administration of sub-toxic doses of 10 and 20 µM *p*-BrBzGSH(Cp)_2_ were used to examine protein levels in GBM cells (Figure 4C). RAGE protein level increased 1.8- and 1.6-fold in T98 cells following 10 or 20 µM *p*-BrBzGSH(Cp)_2_ treatments respectively, whereas in U87 cells RAGE protein levels were not significantly changed following 10 µM *p*-BrBzGSH(Cp)_2,_ but increased 2.5-fold after administration of 20 µM *p*-BrBzGSH(Cp)_2_ (Figure 4C).

Colocalization analyses following immunohistochemical staining for RAGE were used to examine both intracellular distribution and changes in protein expression following treatment with GLO1 inhibitor. Representative analyses are shown for T98 cells prior to and 24 h post-*p*-BrBzGSH(Cp)_2_ administration (Figure 4D). Positive staining for RAGE in the cytoplasm was depicted as red in the inset of Figure 4D, whereas RAGE staining in proximity to the nuclear boundary (presumably associated with the ER) was depicted as yellow. Prior to drug treatment, scattered perinuclear accumulation of RAGE was observed in T98 along with evident cytoplasmic staining. Following *p*-BrBzGSH(Cp)_2_ administration, intense perinuclear staining for RAGE was observed, suggesting up-regulated synthesis of RAGE, most likely a combination of secretory esRAGE and fl-RAGE prior to transit and multimer assembly in the plasma membrane.

### 2.6. shRNA Inhibition of GLO1 in GBM Induces RAGE Expression and Increases DNA-AGEs

**Figure 5:**
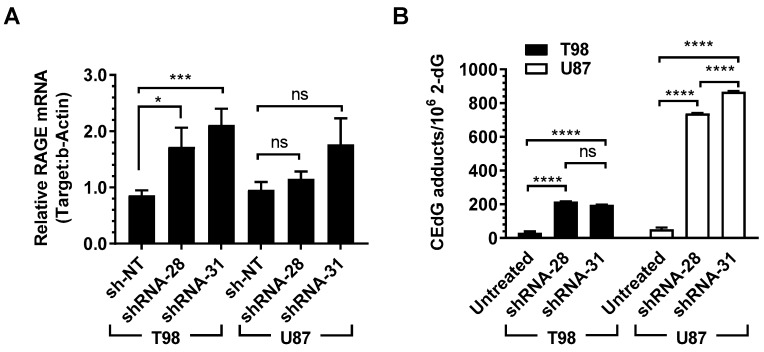
Knockdown of GLO1 induced RAGE expression and increased DNA-AGEs in GBM cells. (**A**) RT-qPCR analysis showed the efficiency of GLO1 knockdown in increasing RAGE transcripts in GBM cells. mRNA expression levels were normalized to *β-*actin and untreated cells, and compared with sh-NT cells. sh-NT: non-target short hairpin RNA (shRNA) virus infected cells; shRNA-28 and shRNA-31: GLO1-shRNA-28 and -31 virus infected cells. Data represent the mean of at least three independent determinations; (**B**) Quantification of (***R***, ***S***) CEdG adducts in untreated or GLO1 knockdown T98 and U87 cells by LC-ESI-MS/MS. Error bars denote the mean ± SEM. Significant differences are indicated (* *p* < 0.05, *** *p* < 0.001, **** *p* < 0.0001, ns = not significant; two-way ANOVA with Tukey’s method to correct for multiple comparisons).

To show that gene expression and protein changes observed upon treatment with *p*-BrBzGSH(Cp)_2_ were the result of GLO1 inhibition and rule out small molecule off-target effects, two GLO1-specific short hairpin RNAs (shRNA-28 and shRNA-31) and a non-targeting shRNA (sh-NT) were introduced into T98 and U87 cells. Because of the strong MG-mediated cytotoxicity of GLO1 inhibition, knockdown T98 clones exhibited severe growth retardation while knockdown U87 clones showed attenuated proliferation. GLO1 and RAGE mRNA levels were measured by RT-qPCR after infection with lentiviral shRNA vectors (Figure 5A). GLO1 mRNA levels decreased modestly following treatment with shRNA-28 and shRNA-31 relative to non-targeting control sh-NT. Stable GLO1 knockdown U87 and T98 clones exhibited reductions of mRNA expression ranging from 38% to 77% relative to the negative controls. Similar levels of GLO1 knockdown using shRNAs was reported by Hosoda et al. [47]. Nevertheless, corresponding effects on RAGE mRNA expression in both cell lines were analogous to those found following GLO1 inhibition with *p*-BrBzGSH(Cp)_2_.

In T98 cells, RAGE mRNA expression increased ~2-fold as a result of GLO1 knockdown (*p* < 0.001; Figure 5A). RAGE was induced to a lesser extent in U87 cells. Stronger expression of RAGE mRNA was observed with the shRNA-31 targeting vector in both cell lines (Figure 5A). Levels of CEdG resulting from GLO1 knockdown in T98 and U87 cells were determined by LC-ESI-MS/MS (Figure 5B). Untreated T98 and U87 cells had similar background levels of CEdG at a frequency of ≤50 per 10^6^ dG. However, GLO1 knockdown by both shRNAs resulted in significant increases in CEdG, with U87 sustaining ~15-fold increase in DNA adduct levels. Significant difference in CEdG levels for shRNA-28 and shRNA-31 was observed in U87 cells (Figure 5B).

### 2.7. Inhibition of GLO1 Abrogates GBM Tumor Growth In Vivo

**Figure 6:**
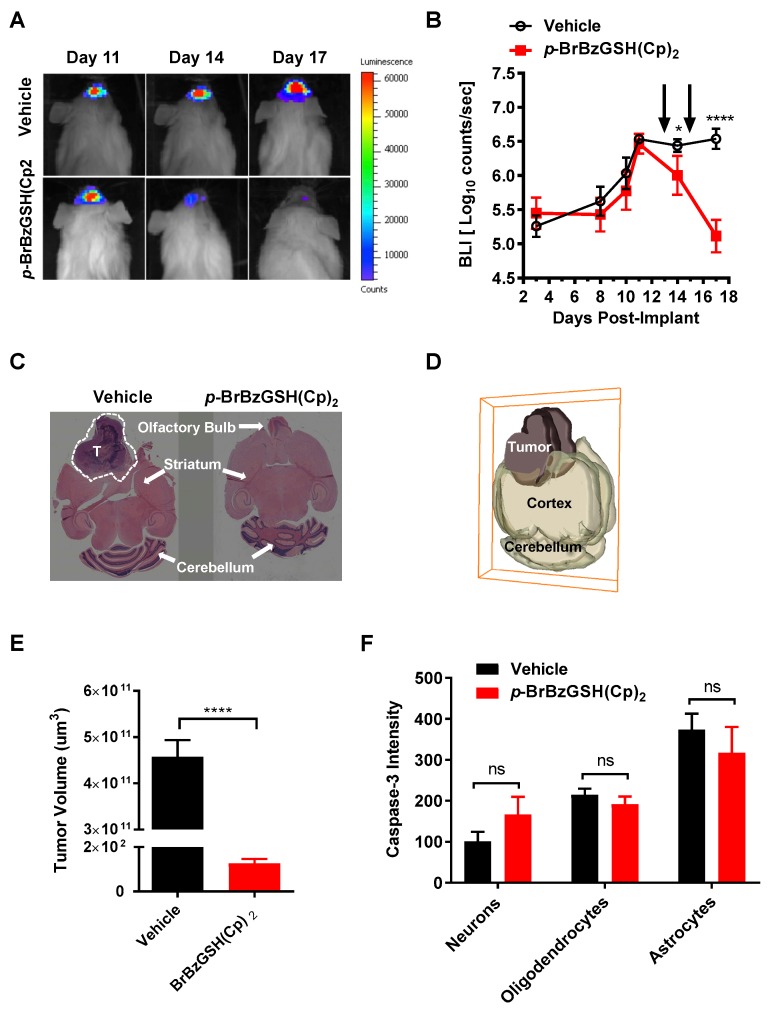
*p*-BrBzGSH(Cp)_2_ decreased GBM tumor volume in mice. (**A**) Representative serial bioluminescence (BLI) acquisition; and (**B**) quantification over 17 days revealed significant reduction in BLI from xenografted GBM tumors in mice injected intraperitoneally with *p*-BrBzGSH(Cp)_2_ vs. vehicle-treated mice (*n =* 12 per group). Data are mean ± SEM, * *p* < 0.05, **** *p* < 0.0001, ns = not significant. Arrows indicate time of drug injections; (**C**) H&E brain slices of vehicle and *p*-BrBzGSH(Cp)_2_-treated mice 17 days post-implant. (**D**) 3D brain reconstruction for measuring tumor volume in vehicle-treated mice 17 days post-implant; (**E**) Tumor volume of vehicle and *p*-BrBzGSH(Cp)_2_ treated mice 17 days post-implant; (**F**) Quantification of comparative immunofluorescence in vehicle and *p*-BrBzGSH(Cp)_2_ treated animals revealed no increase in apoptotic marker Caspase-3 in neurons, oligodendrocytes, and astrocytes.

Proof-of-concept experiments to establish the potential efficacy of GLO1 inhibition for the treatment of GBM were conducted using a mouse xenograft model. U87 glioma cells expressing ZsGreen1-firefly luciferase were used to implant brain tumor xenografts in Non-Obese Diabetic/Severe Combined Immunodeficiency (NOD/SCID) mice (*n =* 24) [48]. In vivo quantification of bioluminescence images (BLI) was used to monitor tumor growth in response to drug or vehicle treatment (Figure 6A). Animals were injected intraperitoneally with *p*-BrBzGSH(Cp)_2_ or vehicle 13 and 15 days after implant, approximately two and four days after maximum tumor growth was established (Figure 6B). BLI data acquired at days 14 and 17 revealed significant diminution of brain tumors in GLO1 inhibitor-treated mice but not in vehicle-treated mice (Figure 6B). At 17 days post-implant, mice were sacrificed and H&E analysis of vehicle-treated mice revealed the presence of large tumors that had damaged the olfactory bulb and extended into the striatum in the frontal cortex (Figure 6C and Figure S7). Three-dimensional quantitative analyses of tumor tissue from the *p*-BrBzGSH(Cp)_2_ treated mice compared to controls confirmed a significant decrease (*p* < 0.0001) in the total tumor volume in drug (1.33 × 10^2^ µm^3^) versus vehicle (4.6 × 10^11^ µm^3^) treated mice (Figure 6D,E).

The potential complication of cytotoxic off-target effects in the surrounding non-tumor brain tissue (neurons, astrocytes, and oligodendrocytes) resulting from GLO1 inhibition was investigated using quantitative immunohistochemistry to assess markers of apoptosis and neural fitness. Comparative studies of vehicle versus *p*-BrBzGSH(Cp)_2_ treated mice did not reveal any statistically significant increase in the apoptotic marker caspase-3 in neurons, oligodendrocytes, or astrocytes (Figure 6F). Additional studies indicated no significant changes in microtubule associated processes (MAP2), a marker of neuronal differentiation, or neuronal nuclear antigen (NeuN), an indicator of neuronal viability, as a result of treatment with *p*-BrBzGSH(Cp)_2_ (Figure S8). There were also no apparent structural changes in myelin basic protein (MBP) produced by oligodendrocytes (Figure S8), indicating intact myelinated axonal fibers. Similarly, we did not observe any change in GFAP+ astrocytes (Figure S8). The vehicle and *p*-BrBzGSH(Cp)_2_ treated mice also maintained relatively stable body weight during our experiments which suggested that there were no obvious signs of general toxicity. Therefore, inhibiting GLO1 abrogated tumor growth in vivo without any discernible off-target effects on surrounding brain cells.

## 3. Discussion

The recognition that many neoplasms rely predominantly on alternative metabolic pathways to meet their energetic demands has led to the idea that specific metabolic targeting could have significant potential for cancer treatment [49]. To protect against the pro-apoptotic effects of MG, glycolytic tumors utilize the glyoxalase system (consisting of GLO1 and GLO2) to convert MG to d-lactate in a two-step transformation [17]. GLO1 (EC 4.4.1.5) catalyzes a 1,2 H-shift of the hemithioacetal formed spontaneously from reaction of MG with glutathione to produce *S*-d-lactoylglutathione. Glyoxalase 2 (EC 3.1.2.6) subsequently hydrolyses *S*-d-lactoylglutathione to regenerate glutathione and liberate D-lactate. Because the major significant chemical transformation of MG occurs during the GLO1 step, inhibition of this enzyme alone is sufficient to prevent the detoxification of MG and induce its accumulation, which may become acute under conditions of elevated glycolytic flux.

The cancerostatic action of MG and its therapeutic potential were initially recognized by Szent-Gyorgi over 40 years ago [50]. However, the chemical reactivity and toxicity of MG precluded any practical therapeutic application when administered systemically. The concept of increasing the local concentration of MG by targeting GLO1 in glycolytic tumors was initially proposed as a potential cancer treatment by Vince and co-workers [35]. Inhibiting GLO1 was predicted to result in the intracellular accumulation of MG leading to the rapid modification of proteins, lipids, and DNA and the induction of cell death by apoptosis [36,51,52]. The suitability of GLO1 targeting for GBM therapy is largely dependent on its expression level relative to normal brain; however, studies of glyoxalase expression in GBMs have been limited and inconsistent. Santarius et al. reported that GLO1 gene amplification is a rare event in gliomas [19], whereas others have found that GLO1 protein levels are elevated in oligodendrogliomas with an intact chromosome 1p (a less aggressive form of brain cancer distinct from GBM) [21]. Although GLO1 overexpression is evident in many tumors, analysis of data from TCGA reveals that this is not universally true. For example, while GLO1 is overexpressed in breast carcinomas, colon adenocarcinomas, and head and neck squamous cell carcinomas, its expression level is lower in kidney clear cell carcinoma, kidney papillary cell carcinoma, and cholangiocarcinoma relative to the corresponding normal tissue (Figure S1). Our own data analysis in Figure 1A revealed significant up-regulation of GLO1 (1.5 fold) in GBM across all subtypes, an effect attributed partly to copy number variation (Figure 1B).

Using quantitative LC-ESI-MS/MS, we observed that inhibition of GLO1 by either a small molecule inhibitor or shRNA targeting in T98 and U87 cell lines caused significant increases in CEdG (Figure 4A and Figure 5B), consistent with increased intracellular accumulation of MG. Because GLO1 is localized to the cytoplasm, increased CEdG levels demonstrated that MG was freely diffusible across the nuclear membrane. Larger increases in CEdG were measured in the more drug-sensitive U87 relative to T98 cells, and these cells also demonstrated increased apoptosis when treated with *p*-BrBzGSH(Cp)_2_ near their respective IC_50_ values of 10 and 100 µM (Figure 3). Prior to drug treatment, background levels of CEdG were similar in both cell lines. We speculate that the increased levels of CEdG in U87 cells induced by GLO1 inhibition contributed to enhanced apoptosis relative to T98.

The lower CEdG levels observed in T98 cells may have been the result of significantly increased GLO1 expression upon treatment with inhibitor (Figure 4B). Although there appeared to be a corresponding increase in U87 cells following administration of *p*-BrBzGSH(Cp)_2_, this was not statistically significant. Increased GLO1 activity may have been the result of elevated MG, an effect which has been previously described for melanoma cells [46]. Reduced induction of CEdG in T98 relative to U87 cells could also reflect enhanced DNA repair, in particular nucleotide excision repair (NER), which has been previously shown to be a major pathway for removal of CEdG [44].

Small molecule inhibition of GLO1 at concentrations near the respective IC_50_ values resulted in a significant increase in fl-RAGE and esRAGE for both cell lines (Figure 4B and Figure S9). After 24 h of inhibitor treatment, RAGE levels were increased to a similar extent in both cell lines. However, in the more drug-resistant T98 cells, GLO1 was significantly up-regulated, whereas this induction was not significant in the U87 line. When T98 cells were titrated with *p*-BrBzGSH(Cp)_2_ at sub-toxic doses (~10 µM), GLO1 mRNA (Figure S9) expression rapidly increased; however, in U87 cells only slight and statistically-insignificant increases were observed near the IC_50_ value. Enhanced GLO1 expression in T98 glioma cells resulted in lower levels of CEdG in DNA (Figure 4A) and likely contributed to greater resistance towards apoptotic cell death induced by *p*-BrBzGSH(Cp)_2_. Inhibition of GLO1 by shRNA mirrored the effects of *p*-BrBzGSH(Cp)_2_ on CEdG levels in both cell lines, with significantly greater DNA damage observed for U87 cells relative to T98 (Figure 5B). The stimulation of GLO1 expression by MG has been previously described in melanoma cells [46], potentially involving an oxidative stress response mediated by an antioxidant response element (ARE) in the 5′ untranslated region of exon 1 [53].

Inhibition of GLO1 using either *p*-BrBzGSH(Cp)_2_ or shRNA induced significant increases in RAGE mRNA (Figure 4B and Figure S9) and protein levels (Figure 4C) in both glioma lines. The induction of RAGE was titratable by *p*-BrBzGSH(Cp)_2_ in T98 cells, with significant increases observed at doses ~1/10 the IC_50_, whereas in the U87 line a significant increase in RAGE protein was only observed near the IC_50_ (Figure 4C). Final levels of RAGE protein were similar. Activation of RAGE in response to GLO1 inhibition was likely mediated by MG accumulation and the subsequent production of AGE signaling molecules. Methylglyoxal has been shown to activate mammalian TORC2 signaling via the Pkc1-Mpk1-MAP kinase cascade with resultant phosphorylation of Akt [54] and induction of NF-κB. In addition to its other well characterized immunostimulatory effects, NF-κB also binds as an enhancer to the RAGE promoter, amplifying its expression resulting in a RAGE cascade [55]. 

Evidence for a protective effect of cRAGE against cytoxicity induced by GLO1 inhibition was provided by measurement of the effect of treatment with the ADAM10 metalloproteinase inhibitor GIX on the *p*-BrBzGSH(Cp)_2_ IC_50_ values (Figure 3B). Pre-incubation with GIX inhibited the cleavage of fl-RAGE at the plasma membrane surface, resulting in a decrease in cRAGE. Since cRAGE possesses the VC1 ligand binding site of fl-RAGE yet lacks the c-terminal domain, it functions as a competitive inhibitor of fl-RAGE signaling. A similar function is performed by esRAGE, produced by alternative splicing rather than by proteolysis of membrane bound fl-RAGE. The increase in sensitivity to *p*-BrBzGSH(Cp)_2_ in both glioma lines via reduction of the competitive inhibitor cRAGE suggests that fl-RAGE signaling via MG induced AGEs or downstream signaling molecules triggers a pro-apoptotic response.

Inhibition of GLO1 strongly induced RAGE protein expression, as shown in the immunohistochemical imaging in Figure 4D. The antibody used in these experiments was not specific for any RAGE isoform, i.e., no discrimination between fl-RAGE and esRAGE was possible, thus staining represents total cytoplasmic RAGE. Intense perinuclear/ER staining (Figure 4D) suggested de novo synthesis of RAGE in response to AGE signaling. RT-qPCR using primers specific for fl-RAGE and esRAGE indicated nearly equivalent mRNA level expression of both isoforms over a range of inhibitor concentrations in both cell lines (Figure S9). Thus, it appears that increased MG and AGEs resulting from GLO1 inhibition can stimulate a coordinated response involving esRAGE, fl-RAGE and cRAGE. The secretion of esRAGE and the maturation of cRAGE from membrane-bound fl-RAGE likely play an important role in attenuating RAGE signaling and reducing the inflammatory response in gliomas, suggesting that potential strategies to limit their production may have therapeutic benefit against GBM.

While the blood–brain barrier (BBB), consisting of endothelial cells, pericytes, and astrocytic end feet, protects the brain from undesirable foreign substances and chemicals in the blood, it also presents an enormous challenge for getting drugs into the brain. In fact, over 95% of drugs do not show useful activity in the brain and many display poor penetration of the BBB [56]. This innate barrier presents a clear obstacle for treating brain tumors. An important and innovative aspect of results from the current work is that *p*-BrBzGSH(Cp)_2_ can readily cross the BBB in mice and exert a selective therapeutic effect in GBM. This observation is consistent with previous work showing that glutathione and several derivatives can cross the BBB [57]. Our data suggest that GLO1 could be a unique and ideal target because it is strongly expressed in GBM tumors. By targeting a tumor-specific adaptation to elevated glycolytic flux, rather than individual components of the glycolysis pathway (e.g., hexokinase, lactate dehydrogenase, etc.), minimal interference with metabolic pathways shared by normal cells can be expected. Consistent with this idea, our in vivo studies showed that *p*-BrBzGSH(Cp)_2_ did not exert detectable toxicity in normal brain cells, as ascertained by caspase activation, suggesting precision targeting of the tumor with minimal off-target effects in surrounding brain tissue (Figure 6F).

Approaches that target altered metabolic adaptations in cancer cells have great potential for clinical companion studies and translation. For example, evidence suggests that overexpression of GLO1 is one mechanism by which chemotherapy can induce multidrug resistance [18,23]. Therefore, the approach of targeting GLO1 could be combined with existing drugs that increase MG levels and pro-apoptotic AGE formation in chemotherapy-resistant tumors. This could be especially germane to managing GBM because temozolomide is the only viable chemotherapeutic strategy and there are no effective second-line therapies for patients that become chemoresistant. Further, the use of GLO1 inhibitors that are systemically deliverable together with the use of existing metabolic neuroimaging for patient selection has the potential for facilitating clinical translation after drug development. One possibility is that patients could be selected for GLO1 inhibition treatment based on MRS tumor profiling and then be monitored for treatment response by pharmacodynamic CEdG metabolite biomarkers quantified by the sensitive LC-ESI-MS/MS method used here.

## 4. Materials and Methods

### 4.1. GLO1 and AGER Gene Expression and the Overall Survival Analyses in Public Datasets

The results are in part based upon data generated by the TCGA Research Network (Available online: http://cancergenome.nih.gov/; February 2015 data version). TCGA Pan-Cancer project RNA-Seq expression data (level 3 data using Illumina HiSeq platform) were applied to the analysis of gene expression levels between primary tumors and normal tissues for different cancer types [26]. Gene-level copy number profile estimated using GISTIC2 algorithm [58] was applied to the analysis of the correlation with gene expression. Log_2_ transformed expression values were used for plotting and statistical analysis. Standard box-plots were used to visualize the expression distribution and differences among different sample types. Statistical significance of the expression differences between groups was determined using Welch’s *t*-test.

To investigate prognostic implications of GLO1 expression, we first queried PROGgeneV2 [27] (Available online: http://www.compbio.iupui.edu/proggene). GLO1 expression cutoffs at either 25, 50 or 75 percentiles were applied to dichotomize patients into high and low expression groups for overall survival analyses across brain cancer datasets. Two of the thirteen datasets, GSE13041 (GBM with Affymetrix U95v2 platform) [28] and GSE4271 (high-grade glioma with Affymetrix U133A platform) [29,30], showed statistically significant differences between GLO1 high and low expression groups based on log-rank tests. To replicate and confirm the findings, we downloaded data of these two datasets from NCBI-GEO (Available online: https://www.ncbi.nlm.nih.gov/geo/). Differences in overall survival between high and low expression groups (using the optimal cut-point derived from the log-rank score tests) were compared by Kaplan–Meier curves using the Survival package in R language environment.

### 4.2. GBM Cell Culture

Media and supplements were obtained from Life Technologies (Carlsbad, CA, USA), unless otherwise indicated. T98 (ATCC CRL-1690) and U87 (ATCC HTB-44) GBM cells were obtained from the ATCC (Manassas, VA, USA) and were cultured in Dulbecco’s modified Eagle’s medium/F12 or DMEM, respectively, supplemented with 10% FBS (Hyclone), Glutamax, and Pen/Strep, cultured on T75 Flasks (NUNC), and maintained at 37 °C and 5% CO_2_. Normal human non-immortalized astrocytes were grown in DMEM/F12 supplemented with N_2_, 2% FBS (Thermo Scientific, Marietta, OH, USA), Glutamax, and Pen/Strep, cultured on T75 Flasks and maintained at 37 °C and 5% CO_2_.

### 4.3. Real-Time PCR (RT-qPCR)

Total RNA was isolated using the Direct-zol™ RNA MiniPrep Kit (Zymo Research, Irvine, CA, USA) according to the manufacturer’s instructions. Each RNA sample (500 ng) was converted to cDNA with the RT^2^ First Strand Kit (Qiagen, Valencia, CA, USA). Real-time PCR was performed using a CFX96 Touch™ Real-Time PCR Detection System (Bio-Rad, Hercules, CA, USA) and the following primers: (i) GLO1: 5′-CCCCAGTACCAAGGATTTTCT-3′, 5′-TGGGAAAATCACATTTTTGGA-3′; (ii) AGER: 5′-CAGACAGAGCCAGGACCC-3′ and 5′-AGCACCCAGGCTCCAACTG-3′; and (iii) ACTB: 5′-CCAACCGCGAGAAGATGA-3′ and 5′-CCAGAGGCGTACAGGGATAG-3′. Primers were designed to measure the expression of GLO1, all 10 transcript variants of RAGE, and β-actin, respectively. Reactions were carried out using the standard cycling parameters per instrument manufacturer recommendations. For each sample, expression levels were normalized to those of β-actin.

### 4.4. Western Blot Analysis

Cells were homogenized in Pierce IP Lysis Buffer containing Halt Protease Inhibitor Cocktail, Halt Phosphatase Inhibitor Cocktail, and 0.5 M EDTA solution (Thermo Fisher Scientific, Waltham, MA, USA). Then 25 µg of total proteins were separated on Mini-PROTEAN TGX Gels (Bio-Rad) and transferred to nitrocellulose membrane (Bio-Rad). Protein bands were detected using GLO1 or RAGE (ABcam, Cambridge, UK) primary antibodies, and super-signal west pico chemiluminescent substrate (Thermo Fisher Scientific) with horseradish peroxidase (HRP)-conjugated secondary antibodies (Cell Signaling, Danvers, MA, USA). The relative intensities of the bands were quantified by scanning densitometry using NIH Image Software (Image J, version 1.46, Available online: http://rsb.info.nih.gov/ij/) and by calculating the average of 3 assays. Each extract was also stained for β-actin to calculate normalized levels.

### 4.5. Patient GBM Tissues and Immunohistochemical (IHC) Staining

Patient tissue samples of glioblastoma multiforme (GBM) were collected in accordance with an approved protocol (IRB# 09062; approved on 1 October 2009) from the City of Hope Institutional Review Board. Tissues were stained as previously described [48]. Briefly, tissue was formalin-fixed paraffin-embedded (FFPE), and stained as follows: Paraffin sections were first dewaxed with xylene and then hydrated with an alcohol gradient (100%, 95%, and 70%). Antigen were recovered by treating sections in Na-Citrate buffer (10 mM, pH 6.0) in a 95 °C water bath for 30 min. Sections were then permeabilized in 0.3% tween-20 for 30 min at 37 °C, and were blocked for non-specific sites by incubating in PBS + 1% bovine serum albumin (BSA) + 10% fetal bovine serum (FBS). For detection, sections were incubated in appropriate primary antibody over night at 4 °C, in PBS + 1.5% FBS + 1% BSA. Primary antibodies were obtained from EMD Millipore Corporation, Billerica, MA, unless otherwise indicated: GFAP, MAP2, NeuN, Caspase 3, GLO1 (Abcam), RAGE (Abcam), and MBP (DAKO, Carpinteria, CA, USA). The next day, sections were incubated with the appropriate IgG secondary antibody conjugated with either Alexa Flour 488, 594, or 647 (Jackson ImmunoResearch Laboratories, Inc., West Grove, PA, USA) in PBS for one hour at room temperature protected from light and then mounted with Immunogold plus DAPI (Thermo Fisher, Pittsburgh, PA, USA). Samples were analyzed on a Carl Zeiss LSM 510 (Carl Zeiss Microscopy, LLC, Thornwood, NY, USA) confocal microscope. Confocal *Z*-stacks were used for colocalization study.

### 4.6. Immunocytochemistry (ICC)

GBM cells (U87, T98) were stained as previously described or treated with *p*-BrBzGSH(Cp)_2_ (10 or 20 µM) for 24 h prior to staining [59]. Briefly, cells were fixed with 4% paraformaldehyde at 37 °C for 10 min. Samples were then blocked for one hour in 1X PBST (Phosphate Buffered Saline with Tween-20) with 1% *w*/*v* non-fat milk. Primary antibodies were diluted in antibody dilution buffer according to manufacturer’s instruction and incubated overnight at 4 °C (GLO1 (ab85420), RAGE (ab37647); Abcam). The next day, samples were incubated with the appropriate IgG secondary antibody conjugated with Alexa Flour 488 (Abcam) for one hour at room temperature protected from light, mounted with Prolong Gold Antifade with DAPI (Thermo Fisher) for confocal imaging. 

### 4.7. Immunofluorescence Intensity Quantification

Samples, Immunohistochemical (IHC) and Immunocytochemistry (ICC), were analyzed on a Carl Zeiss Axio LSM 510 confocal microscope and software *Z*-stacks were acquired for both intensity quantification and colocalization studies. First, tissue or cell sections stained with only secondary fluorescent antibody were imaged to determine detector gain and amplifier offset for threshold signal to reduce false-positivity and tissue auto-fluorescence. Second, confocal *Z*-stacks of test sections, stained with desired primary and secondary antibodies, were imaged under the presets determined from matched control tissue for amplifier offset and detector gain. This insured standardization of measurements and avoidance of fluorescence saturation over the intensity range measured. A minimum of 3 regions of interests per section and subtype were used for intensity measurement and quantification in LSM 510 software (version 4.2).

In colocalization studies, control tissue sections were used to determine detector gain, amplifier offset, and gating through the crosshair function for desired fluorescence channels. Confocal *Z*-stacks of test sections, stained with desired primary and secondary antibodies, were imaged at 0.5 µm intervals under the presets determined from matched control tissue for amplifier offset and detector gain. Crosshair function under the presets determined from matched control tissue was then used to quantify colocalization at 0.5 µm interval.

ICC staining of GBM control or *p*-BrBzGSH(Cp)_2_-treated cells were imaged at the same detector gain and amplifier offset for direct comparison. Fluorescence intensity was analyzed using Image-Pro Premier 9.2 (average integrated optical density, *n* ≥ 50 cells per condition).

### 4.8. 3-D Brain Reconstruction

*Z*-stack images were reconstructed into multi-colored three-dimensional surface renderings using Amira software version 5.4.1 (Visage Imaging GmbH, San Diego, CA, USA). Segmenting multi-channel three-dimensional datasets in thresh-holding mode rendered surfaces.

### 4.9. Cytotoxicity Assay

U87 GBM cells were seeded on 96-well plates at a density of 3.2 × 10^5^ cells/cm^2^ (8000 cells/well). Following 24 h incubation, cells were treated with increasing concentrations of *p-*BrBzGSH(Cp)_2_ as indicated for an additional 24 h. Cellular viability was analyzed using a colorimetric Cell Titer Aqueous Non-Radioactive Cell Proliferation Assay per manufacturer’s instructions (Promega, Madison, WI, USA). Data were plotted using GraphPad Prism 7 (GraphPad Software, Inc., La Jolla, CA, USA) and IC_50_ values calculated using non-linear regression. T98 GBM cells were seeded with collagen on 96-well plates at a density of 3.2 × 10^5^ cells/cm^2^ (8000 cells/well). Cells were treated and analyzed as described for U87 analysis.

### 4.10. Terminal Deoxynucleotidyl Transferase dUTP Nick End Labeling (TUNEL)

GBM cells (T98 and U87) were seeded on coverslips, grown overnight, and treated with 30 µM *p-*BrBzGSH(Cp)_2_ for 24 h. Following cell fixation and permeabilization, the Click-iT^®^ TUNEL-FITC Imaging assay (Life Technologies) was performed according to manufacturer's protocol. Cells were imaged and analyzed using a Carl Zeiss LSM 510 confocal microscope.

### 4.11. shRNA Knockdown of GLO1 Expression

For GLO1 knockdown in T98 and U87 GBM cells, the constructs in pLKO.1 containing GLO1-specific short hairpin RNA (shRNA) sequences directed to human GLO1 mRNA (GenBank/EMBL/DDBJ accession no. NM_006708; shGlo1-28, TRCN0000118628; shGlo1-31, TRCN0000118631) and non-target shRNA control vector (shNT, SHC016) were purchased from Sigma-Aldrich, St. Louis, MO, USA. Briefly, the lentivirus particles were generated by co-transfection of each lentiviral vector and three packaging vectors (pPACKH1 HIV Lentivector Packaging Kit, System Biosciences (SBI), Palo Alto, CA, USA) into 293T cells using Calcium Phosphate transfection method. Two days after transfection, the lentivirus particles (shGlo1-28, shGlo1-31, or shNT) were harvested from the cell culture media and used in the transduction of T98 and U87 GBM cells. Transduced cells were selected with puromycin (Sigma-Aldrich) and the GLO1 expression level following shRNA knockdown was assessed by Real-time PCR analysis.

### 4.12. Isolation of DNA for CEdG Analysis

Cell pellets were re-suspended in tissue lysis buffer containing 100 mM NaCl, 10 mM Tris·Cl, pH 8, 25 mM EDTA, pH 8, 0.5% SDS, 0.2 mg/mL proteinase K, and 10 mM D-penicillamine. For every 10 mg of cells, 120 µL of lysis buffer was used (d-penicillamine was prepared fresh and added immediately prior to lysis). Cell lysis was done overnight in a 50 °C water bath with periodic vortexing. The sample was extracted with an equal volume of phenol/chloroform/isoamyl alcohol (25:24:1). The aqueous fraction was transferred to a new Eppendorf tube and the DNA precipitated by addition of 0.5 volume of 7.5 M ammonium acetate and 2 volumes of 100% ethanol (−20 °C overnight). The DNA was pelleted by centrifugation and washed with 70% ethanol. Pellets were then air-dried before re-suspending with 100 µL of 18.2 mΩ MilliQ water. DNA was quantitated by UV spectrophotometer (Pharmacia/GE Healthcare, Piscataway, NJ, USA).

### 4.13. Preparation of DNA and Quantification of (R, S) CEdG by Mass Spectrometry

Fifty micrograms of genomic DNA was resuspended in 18.2 mΩ MilliQ water and spiked with (***R***, ***S***) ^15^N_5_-CEdG (1.1 pmol) internal standard in a total volume of 100 μL. Samples were digested by denaturing DNA at 95 °C for 5 min, followed by rapid cooling to 4 °C for 5 min, and adjusted to 0.01 M ammonium acetate (pH 5.3). A large excess of nuclease P1 (4 units, Sigma N8630) was added and the mixture was incubated at 37 °C for 12 h. Then, the solution was adjusted to 0.1 M ammonium bicarbonate (pH 7.0), and 0.004 units of Phosphodiesterase I (Sigma P3243) as well as 1 unit of Alkaline phosphatase (New England Biolabs M0290L) were added, and the mix was incubated for an additional 2 h at 37 °C. Under these conditions, gDNA are hydrolyzed essentially into mononucleosides. The DNA hydrolysates were briefly spun down in a bench-top centrifuge and made up to 500 µL with 18.2 mΩ MilliQ water. Samples were then loaded onto Amicon Ultra centrifugal filter with a membrane NMWL of 3 kDa for the removal of enzymes used in hydrolysis according to manufacturer’s instruction (Millipore #UFC 500396, Burlington, MA, USA). Briefly, samples were centrifuged at 14,000× *g* for 30 min with a recovery rate of 15 µL/min. Under this spin condition, the column has ˃90% recovery with a hold-up volume of 48 µL. The eluent was dried in a speed-vac and reconstituted in 100 µL of LC/MS grade H_2_O (Fluka Analytical #39253-4L-R). A portion of the reconstituted enzymatic hydrolysate (25 µL) was taken and diluted two-fold with 18.2 mΩ MilliQ water for HPLC-DAD quantitation of 2′-deoxyguanosine (dG). The remainder of the hydrolysate was stored at −80 °C for CEdG analysis. CEdG diastereomers were quantified by LC-ESI-MS/MS in an Agilent 6490 tandem mass spectrometer using the stable isotope dilution method as previously described [25].

### 4.14. GBM Xenograft Assay

The ZsGreen-firefly luciferase construct was generated by cloning the *luc2* gene (firefly luciferase) into the EcoRI and BamHI sites of pLVX-IRES-ZsGreen1 vector (Clontech, Mountain View, CA, USA). The construct was used to transfect U87 cells. The firefly luciferase expression is under the control of the CMV promoter. U87 glioma cells expressing ZsGreen-firefly luciferase were suspended in PBS at 10^5^ cells/2 µL, and were injected 2 mm right and 1 mm anterior to the bregma suture of NOD/SCID mice (12 mice/group, total = 24) in accordance with approved City of Hope IACUC protocol (IACUC# 10044; approved in May 2011). Mice were injected intraperitoneally with 10 µL of *p*-BrBzGSH(Cp)_2_ (50mg/kg) or vehicle (0.3% tween-80/PBS) at 13 and 15 days post-implant. In vivo bioluminescence imaging (BLI) was performed with the Xenogen System and Living Image software for data acquisition (Caliper Life Sciences, Hopkinton, MA, USA) following intraperitoneal injection with 3 mg d-Luciferin (Promega).

### 4.15. Statistical Analyses

Unless indicated otherwise, data are represented as mean values ± standard error of the mean. All experimental datasets passed normality test and showed no significant deviation from normal distribution. Statistical significance was assessed using Student’s *t*-test and 1-way ANOVA with Bonferroni’s multiple comparison test or 2-way ANOVA with Tukey’s method when correcting for multiple comparisons (*p*-value: * *p* < 0.05, ** *p* < 0.01, *** *p* < 0.001, and **** *p* < 0.0001; 95% Confidence interval of difference).

## Supplementary Materials

Supplementary materials can be found at http://www.mdpi.com/1422-0067/19/2/406/s1.

## Abbreviations

AGEs	advanced glycation endproducts
CEdG	*N*^2^-1-(carboxyethyl)-2′-deoxyguanosine
3-DG	3-deoxyglucosone
^18^FDG	2-^18^fluoro-2′-deoxy-d-glucose
GBM	glioblastoma multiforme
GFAP	glial fibrillary acidic protein
GLO1	glyoxalase 1
GLUT1	glucose transporter 1
GSH	glutathione
^1^H-MRS	proton magnetic resonance spectroscopy
LC-ESI-MS/MS	liquid chromatography electrospray ionization tandem mass spectrometry
MAP2	microtubule associated processes
MBP	myelin basic protein
MG	methylglyoxal
mRNA	messenger RNA
NeuN	neuronal nuclear antigen
NOD/SCID	Non-Obese Diabetic/Severe Combined Immunodeficiency
*p-*BrBzGSH(Cp)_2_	*S*-(*p*-bromobenzyl) glutathione dicyclopentyl ester
PET	positron emission tomography
RAGE	Receptor for advanced glycation endproducts
esRAGE	endogenous secretory form of RAGE
fl-RAGE	full-length form of RAGE
sRAGE	soluble form of RAGE
RT-qPCR	real-time reverse transcription polymerase chain reaction
shRNA	short hairpin RNA
TCGA	The Cancer Genome Atlas
TUNEL	terminal deoxynucleotidyl transferase dUTP nick end-labeling
