# Supplementary material for: Inhibition of GLO1 in Glioblastoma Multiforme Increases DNA-AGEs, Stimulates RAGE Expression, and Inhibits Brain Tumor Growth in Orthotopic Mouse Models
Source: Int J Mol Sci. 2018 Jan 30;19(2):406. doi: 10.3390/ijms19020406 (PMC5855628; doi:10.3390/ijms19020406)
Supplement: Supplementary file 1 [file ijms-19-00406-s001.pdf]

Supplementary Material for

Inhibition of GLO1 in glioblastoma multiforme increases DNA-AGEs, stimulates RAGE expression, and inhibits brain tumor growth in orthotopic mouse models.

Rahul Jandial[1], Josh Neman[1,5], Punnajit P. Lim[2], Daniel Tamae[2,4,6], Claudia M. Kowolik[2], Gerald E. Wuenschell[2], Sarah C. Shuck[2], Alexandra K. Ciminera[2,4], Luis R. De Jesus[2,7], Ching Ouyang[3], Mike Y. Chen[1], and John Termini[2*]

1 Division of Neurosurgery, [2]Department of Molecular Medicine, [3]Center for Informatics, [4]Irell and Manella Graduate School of Biological Sciences, City of Hope & Beckman Research Institute, 1500 East Duarte Road, Duarte, CA 91010; rjandial@coh.org (R.J.); plim@coh.org (P.L.); ckowolik@coh.org (C.K.); gwuenschell@coh.org (G.W.); sshuck@coh.org (S.S.); aciminera@coh.org (A.C.); couyang@coh.org (C.O.); mchen@coh.org (M.C.)

5 Department of Neurosurgery, Keck School Medicine, University of Southern California, 1975 Zonal Ave, Los Angeles, CA 90033; yebrahim@usc.edu (J.N.)

6 Department of Chemistry and Biochemistry, California State University, Northridge, 18111 Nordhoff Street, Northridge, CA 91330; daniel.tamae@csun.edu (D.T.)

7 Department of Chemistry, Texas A&M University, PO Box 30012, College Station, TX 77842; luis.dejesus@chem.tamu.edu (L.D.J.)

* Correspondence: jtermini@coh.org (J.T.); Tel.: 626-218-8169; Fax: 626-930-5330

Figure S1: GLO1 expression in various cancer types. TCGA Pan-Cancer project RNA-Seq expression data (level 3 data using Illumina HiSeq platform) were applied to the analysis of GLO1 gene expression levels between primary tumors and normal tissues for different cancer types. Sixteen cancer types having data for both primary tumors and normal tissues ($N \geq 5$) were included in the analysis. Ten of the sixteen cancer types investigated, including GBM, showed statistically significant up-regulation of GLO1 expression. Standard box-plots were applied to visualize the expression distribution for each sample type. The red dots show the average values of each distribution. Fold changes (FC) in gene expression and number of samples (N) are indicated at the bottom. Statistical p-values between groups were calculated using Welch's t-test; ***p<0.001, **p < 0.01, *p < 0.05, n.s.: not significant. Legend keys for the abbreviated cancer types: BLCA: bladder urothelial carcinoma; BRCA: breast invasive carcinoma; CHOL: cholangiocarcinoma; COAD: colon adenocarcinoma; GBM: glioblastoma multiforme; HNSC: head and neck squamous cell carcinoma; KICH: kidney chromophobe; KIRC: kidney renal clear cell carcinoma; KIRP: kidney renal papillary cell carcinoma; LIHC: liver hepatocellular carcinoma; LUAD: lung adenocarcinoma; LUSC: lung squamous cell carcinoma; PRAD: prostate adenocarcinoma; READ: rectal adenocarcinoma; THCA: thyroid carcinoma; UCEC: uterine corpus endometrial carcinoma. GBM (also shown in Figure 1) is boxed blue.

Figure S2: Overall survival analysis based on GLO1 expression. Kaplan-Meier analyses of the overall survival in two brain cancer datasets based on GLO1 expression (Panel A: GSE13041 – GBM with

Affymetrix U95v2 platform; Panel B: GSE4271 – high-grade glioma with Affymetrix U133A platform). The analyses employ log-rank score tests to identify the optimal cut-point for best separation of patient groups. The grey dotted line shows the 3-year mark. The log-rank test *p*-value is shown in each panel.

Figure S3: RAGE (AGER) expression in various cancer types. Same as Figure S1, RAGE gene expression levels between primary tumors and normal tissues for different cancer types from TCGA Pan-Cancer project RNA-Seq data were delineated. Eight of the sixteen cancer types investigated, including GBM, showed statistically significant up-regulation of AGER expression.

Figure S4: Representative histological sections of patient GBM tissue obtained after surgery and stained for tumor stroma using hematoxylin-eosin (H&E) which revealed scattered pleomorphic nuclei consistent with the pathological criteria for malignancy.

Figure S5: Expression of GLO1 and RAGE in newly obtained human GBM specimens. Immunofluorescence in patient GBM tissues revealed strong GLO1 and RAGE expression (green). GFAP staining (red) indicates tumors of glial origin. Nuclei were stained with DAPI (blue). Hematoxylin-eosin (H&E) was used to stain for tumor stroma which revealed scattered pleomorphic nuclei consistent with the pathological criteria for malignancy. Scale bars represent 40 μm.

Figure S6: LC-ESI-MS/MS chromatogram of (*R*,*S*) CEdG. Representative MS/MS chromatograms in multiple reaction mode (MRM) for measurement of CEdG in nuclear DNA. Top panel, natural abundance mass transitions at m/z 340→224 for ***R*** (~2 m) and ***S*** (~2.4 m) stereoisomers of CEdG from T98 cells; bottom panel, isotopically enriched mass transitions for ***R*** and ***S*** CEdG at m/z 345→229.

Figure S7: H&E brain slice of *p*-BrBzGSH(Cp)$_2$-treated mice 17 days post-implant. Hematoxylin-eosin (H&E) was used to stain for tumor stroma and identify the tumor region (dotted white line; also shown in Figure 6C).

Figure S8: GLO1 inhibition shows no toxicity in normal brain cells. Immunofluorescence in (A) vehicle and (B) *p*-BrBzGSH(Cp)$_2$ treated animals revealed no increase in apoptotic marker Caspase-3 in neurons (MAP2$^+$/NeuN$^+$), astrocytes (GFAP$^+$), and oligodendrocytes (MBP$^+$).

Figure S9: *p*-BrBzGSH(Cp)$_2$ treatment induces expression of GLO1 and RAGE. Inhibition of GLO1 with *S*-(*p*-bromobenzyl) glutathione dicyclopentyl ester [*p*-BrBzGSH(Cp)$_2$] elevated expression of GLO1 and various isoforms of the receptor for AGE (RAGE), esRAGE and fl-RAGE.

Figure S1.

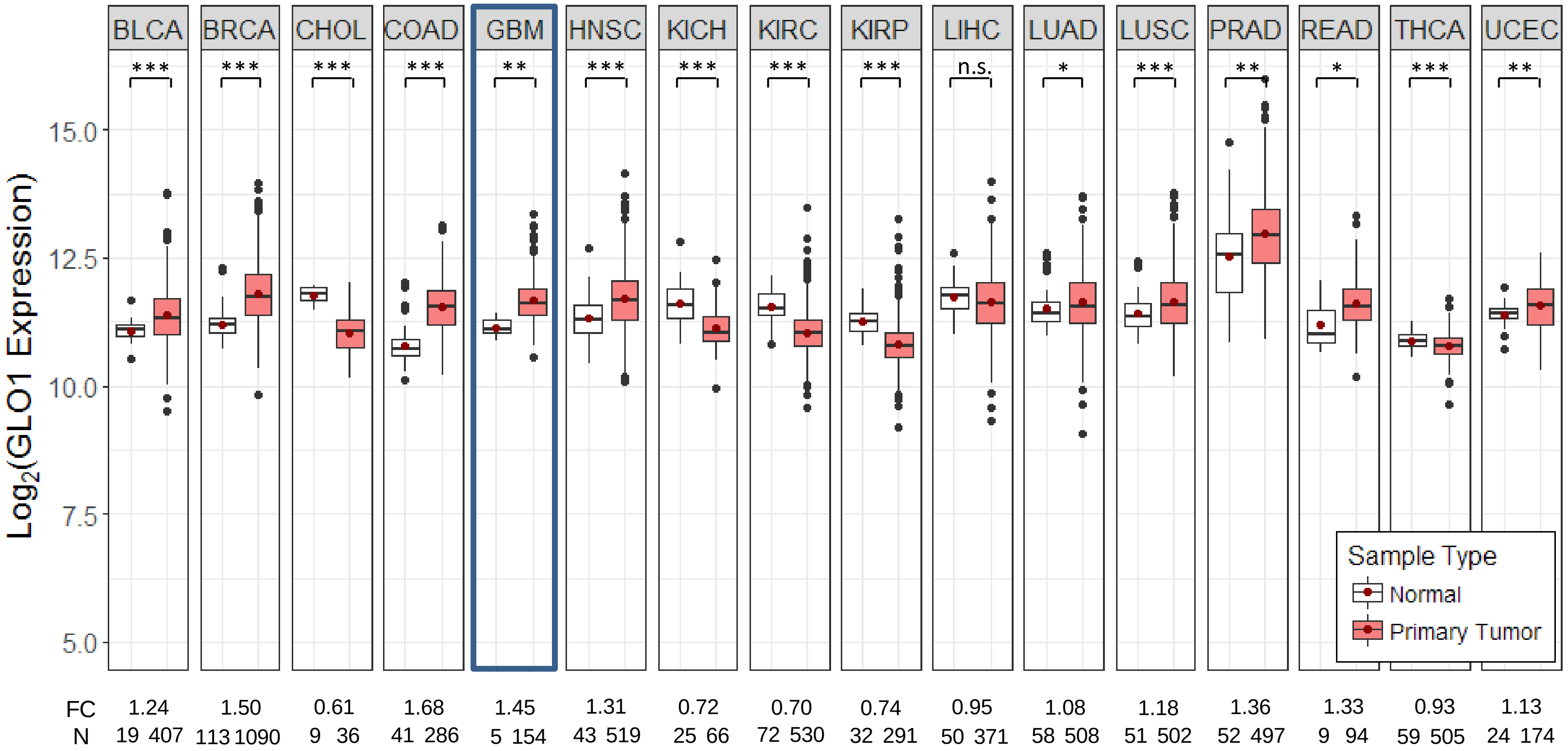
BLCA
BRCA
CHOL
COAD
GBM
HNSC
KICH
KIRC
KIRP
LIHC
LUAD
LUSC
PRAD
READ
THCA
UCEC
Log2(GLO1 Expression)
15.0
12.5
10.0
7.5
5.0
***
**
*
n.s.
Sample Type
Normal
Primary Tumor
FC
N
1.24
19 407
1.50
113 1090
0.61
9 36
1.68
41 286
1.45
5 154
1.31
43 519
0.72
25 66
0.70
72 530
0.74
32 291
0.95
50 371
1.08
58 508
1.18
51 502
1.36
52 497
1.33
9 94
0.93
59 505
1.13
24 174

Figure S2

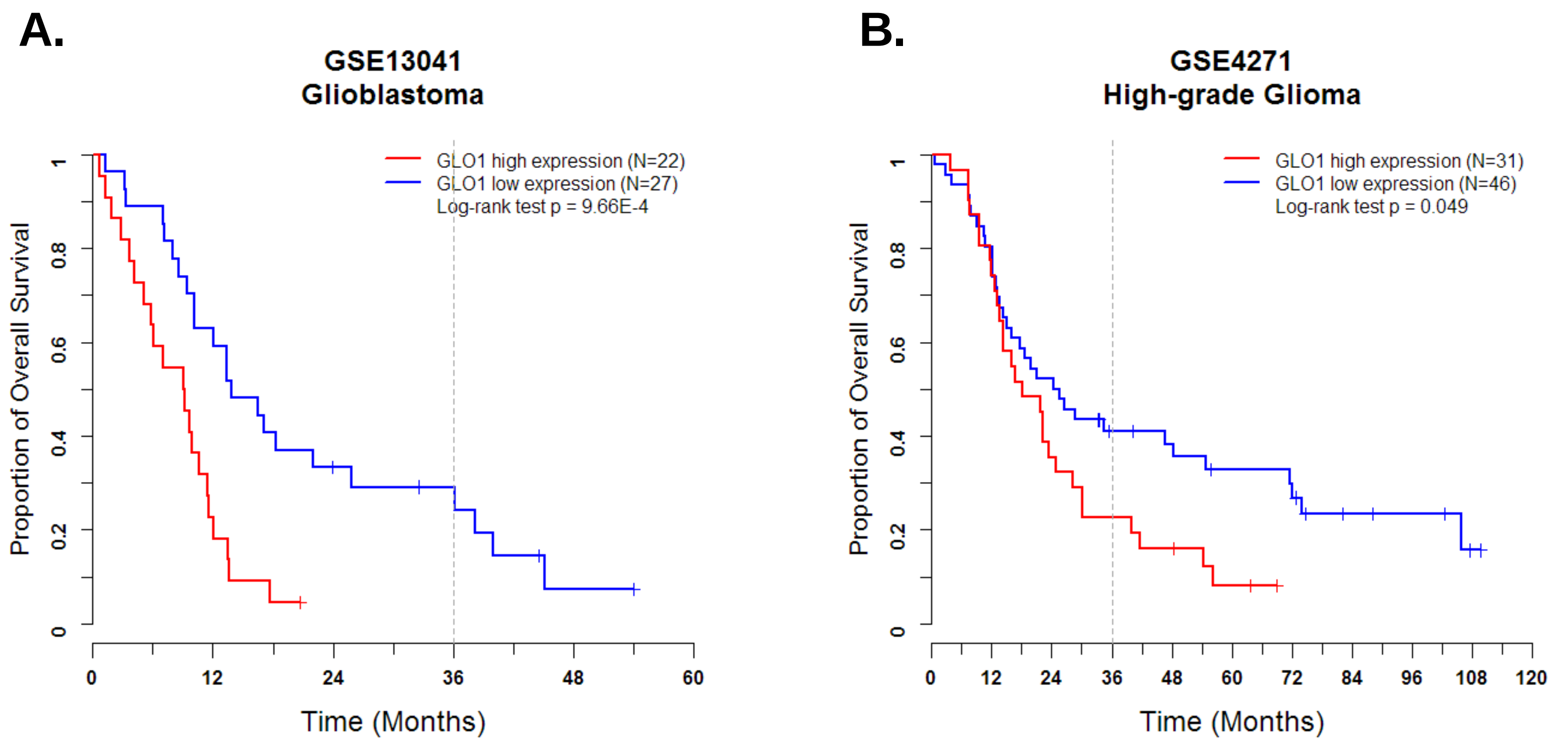
A.
GSE13041
Glioblastoma
GLO1 high expression (N=22)
GLO1 low expression (N=27)
Log-rank test p = 9.66E-4
Proportion of Overall Survival
0
0.2
0.4
0.6
0.8
1
0
12
24
36
48
60
Time (Months)
B.
GSE4271
High-grade Glioma
GLO1 high expression (N=31)
GLO1 low expression (N=46)
Log-rank test p = 0.049
Proportion of Overall Survival
0
0.2
0.4
0.6
0.8
1
0
12
24
36
48
60
72
84
96
108
120
Time (Months)

Figure S3.

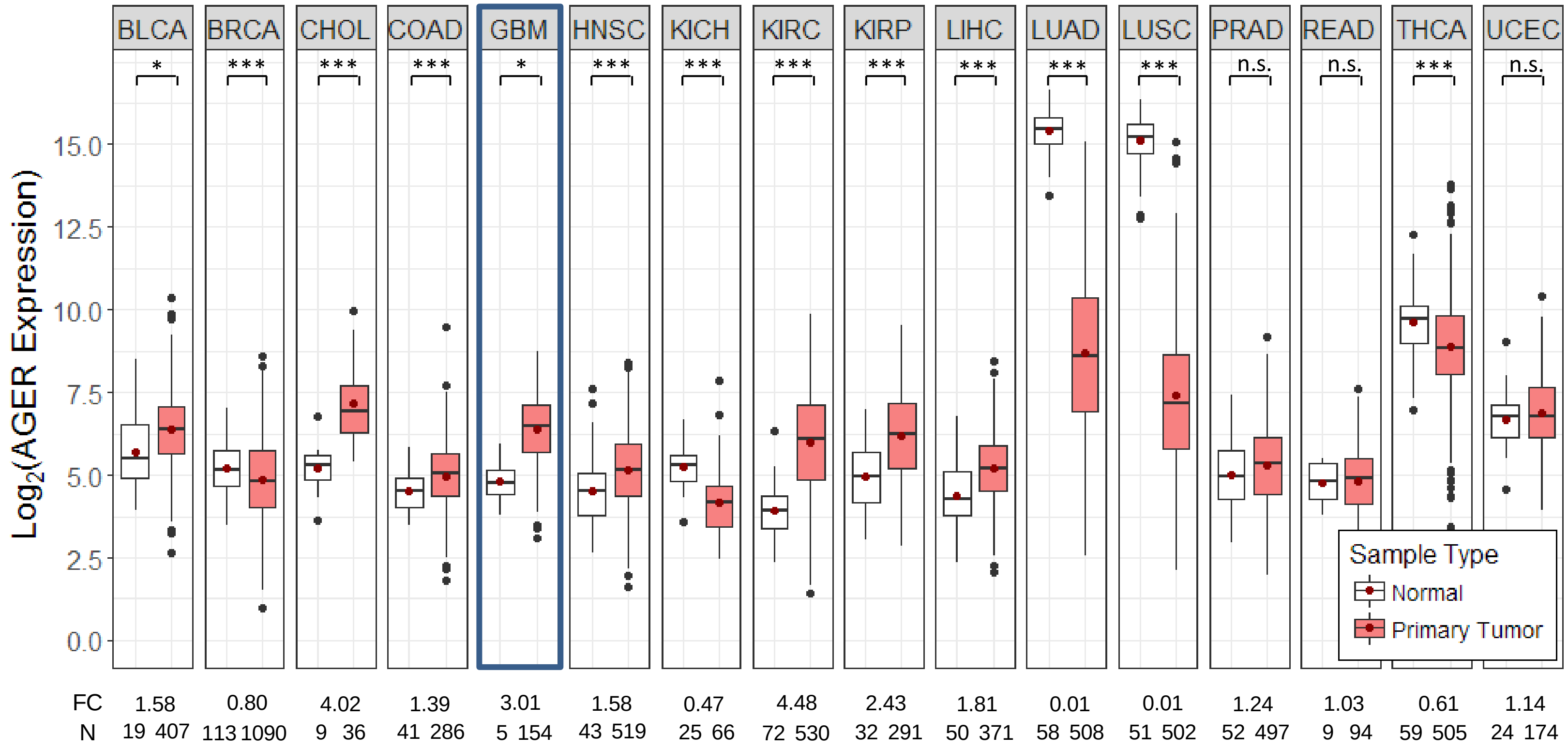

BLCA
BRCA
CHOL
COAD
GBM
HNSC
KICH
KIRC
KIRP
LIHC
LUAD
LUSC
PRAD
READ
THCA
UCEC
*
***
***
***
*
***
***
***
***
***
***
***
n.s.
n.s.
***
n.s.
15.0
12.5
10.0
7.5
5.0
2.5
0.0
Log2(AGER Expression)
Sample Type
Normal
Primary Tumor
FC 1.58 0.80 4.02 1.39 3.01 1.58 0.47 4.48 2.43 1.81 0.01 0.01 1.24 1.03 0.61 1.14
N 19 407 113 1090 9 36 41 286 5 154 43 519 25 66 72 530 32 291 50 371 58 508 51 502 52 497 9 94 59 505 24 174

Figure S4.

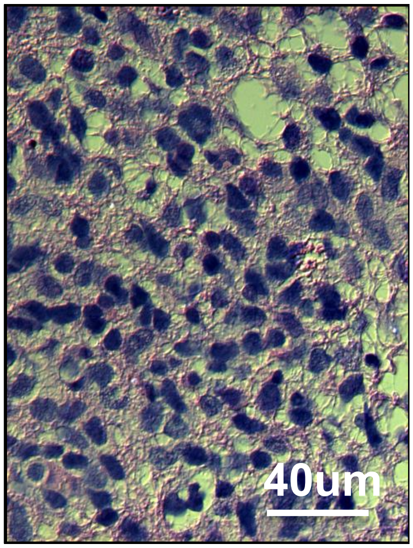

Figure S5.

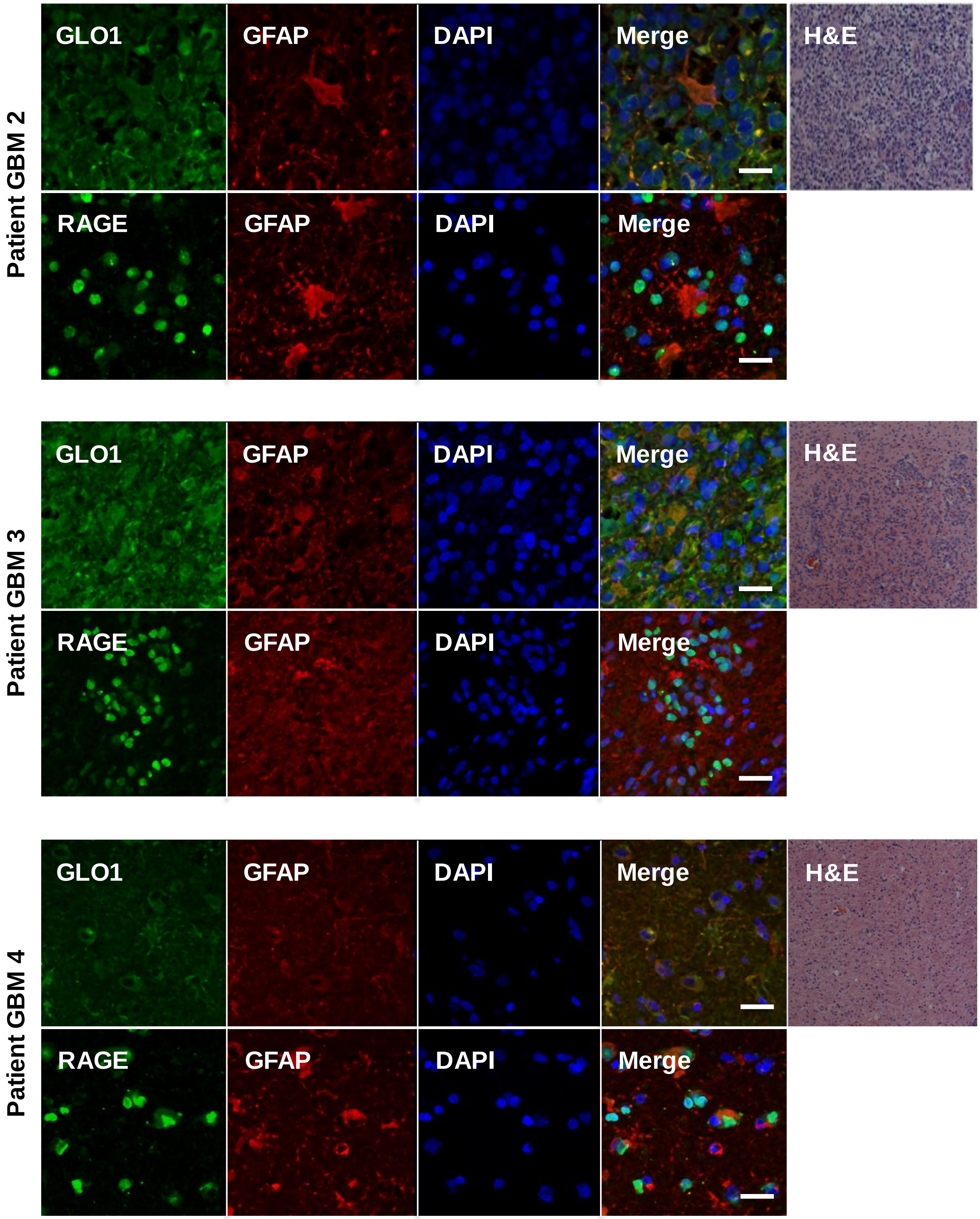
Patient GBM 2
GLO1
GFAP
DAPI
Merge
H&E
RAGE
GFAP
DAPI
Merge
Patient GBM 3
GLO1
GFAP
DAPI
Merge
H&E
RAGE
GFAP
DAPI
Merge
Patient GBM 4
GLO1
GFAP
DAPI
Merge
H&E
RAGE
GFAP
DAPI
Merge

Figure S6.

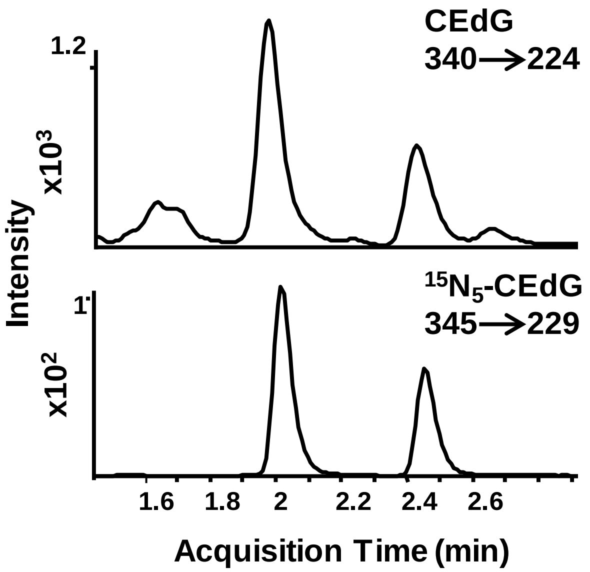

Figure S7

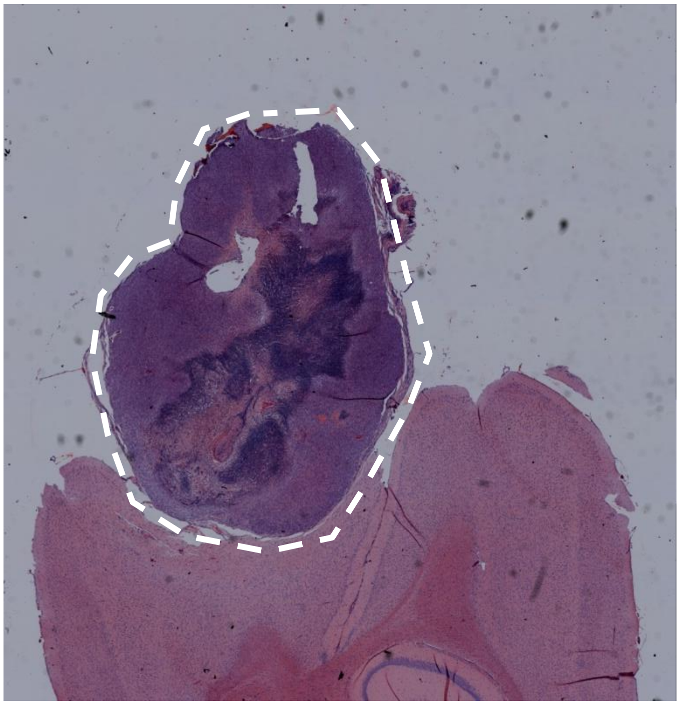

Figure S8.

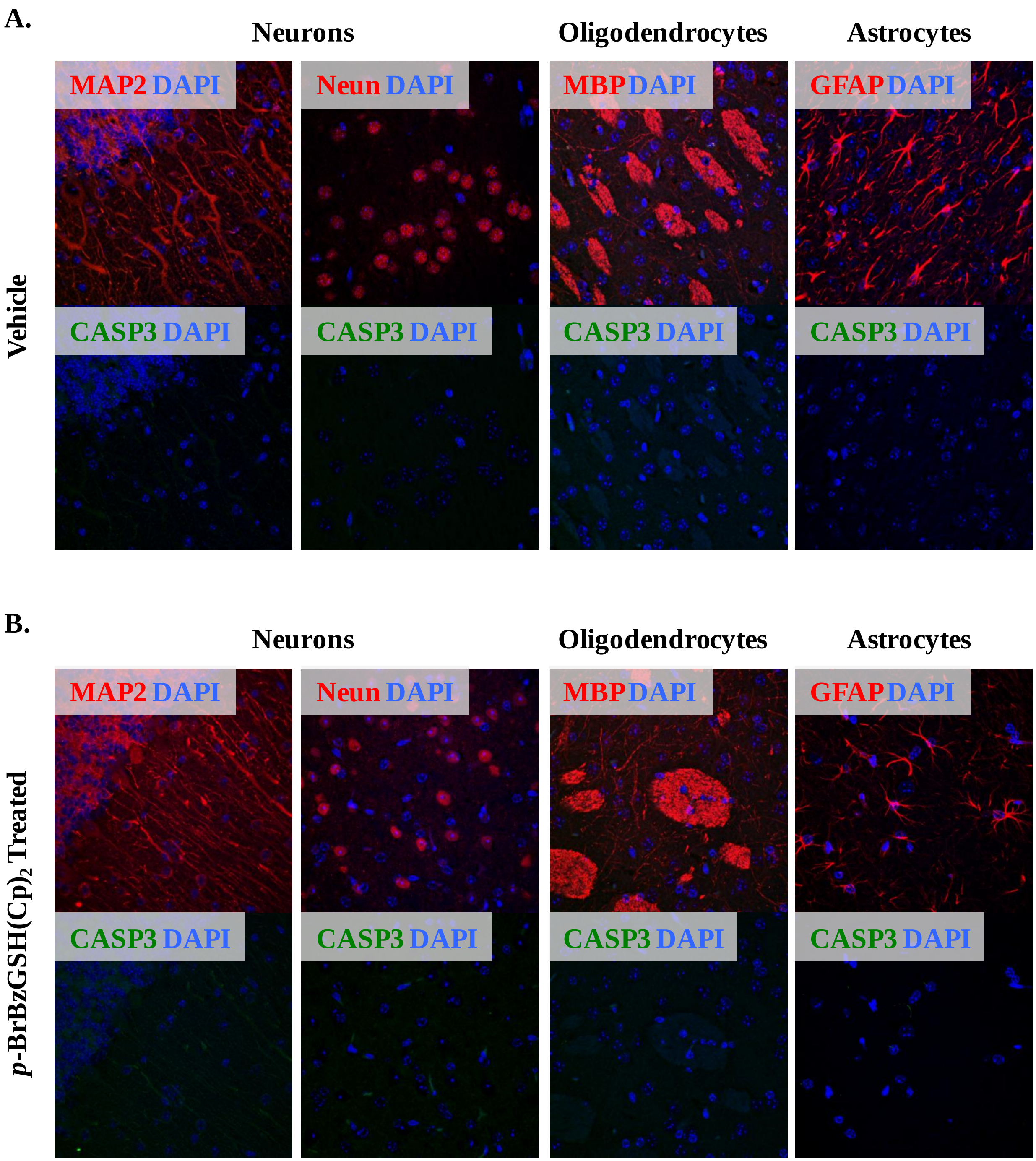
A.
Neurons
Oligodendrocytes
Astrocytes
Vehicle
MAP2 DAPI
Neun DAPI
MBP DAPI
GFAP DAPI
CASP3 DAPI
CASP3 DAPI
CASP3 DAPI
CASP3 DAPI
B.
Neurons
Oligodendrocytes
Astrocytes
p-BrBzGSH(Cp)2 Treated
MAP2 DAPI
Neun DAPI
MBP DAPI
GFAP DAPI
CASP3 DAPI
CASP3 DAPI
CASP3 DAPI
CASP3 DAPI

Figure S9.

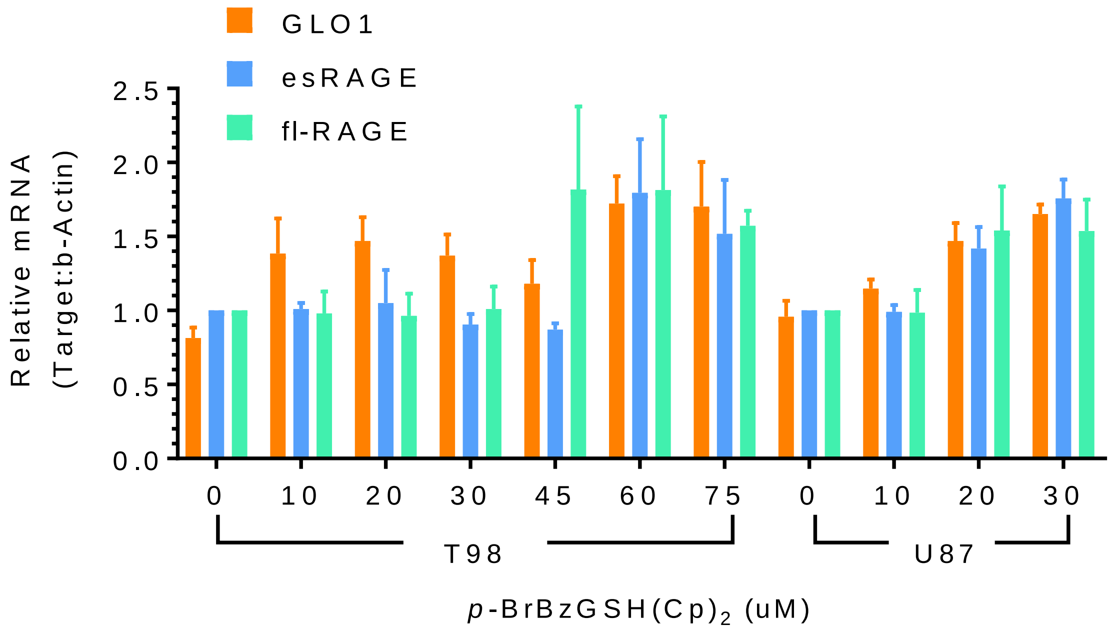
GLO1
esRAGE
fl-RAGE
Relative mRNA (Target:b-Actin)
2.5
2.0
1.5
1.0
0.5
0.0
0
10
20
30
45
60
75
0
10
20
30
T98
U87
p-BrBzGSH(Cp)2 (uM)
